# Soil contamination by *Echinococcus multilocularis* in rural and urban vegetable gardens in relation to fox, cat and dog faecal deposits

**DOI:** 10.1051/parasite/2021073

**Published:** 2021-11-01

**Authors:** Abdou Malik Da Silva, Matthieu Bastien, Gérald Umhang, Franck Boué, Vanessa Bastid, Jean-Marc Boucher, Christophe Caillot, Carine Peytavin de Garam, Camille Renault, Marine Faisse, Sandra Courquet, Vincent Scalabrino, Laurence Millon, Jenny Knapp, Marie-Lazarine Poulle

**Affiliations:** 1 Chrono-Environnement, UMR UBFC/CNRS 6249 Aff. INRA, University of Bourgogne Franche-Comté 25030 Besançon France; 2 Department of Parasitology–Mycology, National Reference Centre for Echinococcoses, University Hospital of Besançon 25030 Besançon France; 3 Entente for the Control of Zoonoses Malzéville 54220 Nancy France; 4 Epidémio-Surveillance et Circulation des Parasites dans les Environnements (ESCAPE), EA 7510, CAP SANTE, Université de Reims Champagne Ardenne 51095 Reims Cedex France; 5 ANSES Nancy Laboratory for Rabies and Wildlife, National Reference Laboratory for Echinococcus spp., Wildlife Surveillance and Eco-Epidemiology Unit, Technopole Agricole et Vétérinaire 54220 Malzéville France; 6 CERFE, Université de Reims Champagne-Ardenne 08240 Boult-aux-Bois France

**Keywords:** Environmental contamination, Soil-transmitted parasites, Foodborne parasites, Copro-qPCR, Soil flotation

## Abstract

*Echinococcus multilocularis* eggs are deposited on the ground with the faeces of the carnivore definitive hosts. A reliable assessment of the spatial distribution of *E. multilocularis* eggs in environments used by humans is crucial for the prevention of alveolar echinococcosis (AE). This study was conducted in 192 rural and 71 urban vegetable gardens in AE endemic areas of north-eastern France. Its objective was to explore the relationship between the spatial distribution of *E. multilocularis* estimated from the collection and molecular analysis of two types of samples: faeces and soil. A total of 1024 carnivore faeces and 463 soil samples were collected and analysed by real-time PCR. No fox droppings and no positive soil samples were collected from the urban gardens. Positive soil samples, positive carnivore faeces, or both, were found in 42%, 24% and 6% of the sampled rural gardens, respectively. No significant association was found between the detection of *E. multilocularis* in soil samples collected from 50 gardens during a single sampling session and the extent and frequency of deposits of fox and cat faeces collected during repeated sampling sessions conducted in the previous months. In 19/50 gardens, *E. multilocularis* was detected in the soil while no positive faeces had been collected in the previous 12 months. Conversely, in 8/50 gardens, no soil samples were positive although positive faeces had been collected in the previous months. Collecting and analysing faeces provide information on soil contamination at a given time, while analysing soil samples provides an overview of long-term contamination.

## Abstract (fr)

Les œufs d’*Echinococcus multilocularis* sont déposés sur le sol avec les fèces des carnivores hôtes définitifs. Une évaluation fiable de la distribution spatiale des œufs d’*E. multilocularis* dans les environnements utilisés par l’homme est cruciale pour la prévention de l’échinococcose alvéolaire (EA). La présente étude a été conduite dans 192 jardins potagers ruraux et 71 jardins potagers urbains des zones endémiques d’EA du nord-est de la France. Son objectif était d’explorer la relation entre la distribution spatiale d’*E. multilocularis* estimée à partir de la collecte et de l’analyse moléculaire de deux types d’échantillons : des fèces et du sol. Au total, 1024 fèces et 463 échantillons de sol ont été collectés et analysés par PCR en temps réel. Aucun excrément de renard et aucun échantillon de sol positif n’a été collecté dans les jardins urbains. Des échantillons de sol positifs, des fèces de carnivores positives ou les deux ont été trouvés dans 42 %, 24 % et 6 % des jardins ruraux échantillonnés. Aucune association significative n’a été trouvée entre la détection d’*E. multilocularis* dans les échantillons de sol collectés dans 50 potagers lors d’une unique session d’échantillonnage et l’importance et la fréquence des dépôts de fèces de renards et de chats collectées lors d’échantillonnages répétés conduits au cours des mois précédents. Dans 19/50 potagers, *E. multilocularis* a été détecté dans le sol alors qu’aucun excrément positif n’avait été collectés dans les 12 mois précédents. A l’inverse, dans 8/50 potagers aucun échantillon de sol n’était positif alors que des fèces positives avait été collectées dans les mois précédents. La collecte et l’analyse de fèces renseignent sur la contamination du sol à un instant donné, alors que l’analyse d’échantillons de sol fournissent un aperçu de la contamination à long terme.

## Introduction

Soil is an important transmission route for zoonotic parasites such as helminths or protozoa whose infective eggs, oocysts or larvae are spread in the environment with faeces of foxes, dogs and cats [2, 44, 48, 66]. Soil types, moisture, and local conditions influence the distribution of these zoonotic agents [1, 12, 62, 68]. Putting soiled hands in the mouth as well as geophagia are identified as risk factors for these soil-transmitted parasite zoonoses [7, 37, 38, 66]. As a consequence, contamination of sandboxes, backyards, public parks or beaches with zoonotic parasites is a public health concern [4, 18, 35, 55, 66]. The consumption of raw fresh fruit and vegetables in contact with soil contaminated with foodborne parasites is also increasingly recognized as a transmission route of zoonotic diseases [6, 17, 38, 41, 51, 52, 69]. However, most people are unaware of the risk of zoonotic diseases from intestinal parasites of carnivores [39, 57, 65] and few studies have focused on the parasitic contamination of vegetable gardens [4, 27, 32, 41]. In some vegetable gardens of north-eastern France, a high density of fox and cat faeces was found, and a large part of the collected faeces tested positive for zoonotic parasites [4, 5, 43]. The risk of human exposure through consumption of raw fruit and vegetables grown in contaminated soil is of particular concern with regard to *Echinococcus multilocularis* (Leuckart, 1863), the cestode responsible for alveolar echinococcosis (AE), a rare but severe and sometimes fatal human disease [9, 64]. Human infection can occur after accidental ingestion of *Echinococcus multilocularis* eggs deposited on the ground with the faeces of infected definitive hosts. These eggs can survive for more than a year in cold and damp conditions [71].

The red fox, *Vulpes vulpes*, is the main definitive host of *E. multilocularis* in Europe and is responsible for most of the environmental contamination with *E. multilocularis* eggs in both urban and rural areas [25]. In the fox population, a low proportion of individuals, mostly juveniles, harbour almost the entire parasitic biomass with the possibility of simultaneous and successive re-infections [20, 46, 49, 60]. As juvenile foxes are likely to disperse, the risk of spreading their pathogens is high [22, 23, 58]. The domestic dog, *Canis lupus familiaris*, is also a definitive host of *E. multilocularis* and its contribution to AE transmission may be significant. The low prevalence of *E. multilocularis* in the dog population is compensated for by the high density of dog faeces around human settlements, their high proximity with humans and a high biotic potential of the parasite in the dog [25, 26, 31, 34]. In addition, experimental infections showed that the domestic cat, *Felis silvestris catus* can harbour the adult worms of *E. multilocularis* but generally with low mature worm burden, excreting few eggs which have not yet been proven to be infective [26, 63, 67]. However, *E. multilocularis* eggs have been found in cat faeces collected in the field [15, 29, 40]. The parasite is usually aggregated in a few micro-foci [15, 31, 43, 47, 70] where distribution in the urban and rural human environments is a health concern in AE endemic regions [16, 20, 31, 33, 50]. Reliable information on the distribution of locations where *E. multilocularis* eggs are aggregated in the soil is crucial for AE prevention.

Assessment of environmental contamination with *E. multilocularis* eggs is often based on carnivore faeces sampling coupled with molecular analysis of faecal samples [14, 31, 43]. The development of PCR or real-time PCR has allowed for very sensitive and rapid detection of both host species DNA and parasite DNA in faeces [28, 30, 36]. In addition, the detection of *E. multilocularis* in soil samples is now possible thanks to the recent development of sensitive and reliable flotation/filtration techniques combined with molecular biology [59, 68]. However, while the collection of a single *E. multilocularis* PCR-positive scat is sufficient to suggest that the soil is contaminated with this parasite, the absence of such faeces at the moment of collection does not necessarily mean that the soil is free of *E. multilocularis* eggs (see e.g., results by Umhang et al. [68]). As the probability of detecting carnivore faeces in the field depends on the species, weather conditions, substrate type and carnivore diet [3, 21, 54], the observed distribution of collected faeces may not be fully representative of their deposit. Furthermore, *Echinococcus multilocularis* eggs may have persisted for weeks or months after the faeces that carried them have disappeared. They may also have been dispersed far from their place of deposit by runoff [62] or soil ploughing.

Our objective was to explore the relationship between environmental contamination by *E. multilocularis* eggs, classically assessed by the collection and molecular analysis of carnivore faeces, and contamination assessed by the collection and molecular analysis of soil samples. The study was conducted in AE endemic areas of north-eastern France and focused on rural and urban vegetable gardens. Specifically, we estimated whether the detection of *E. multilocularis* in a small number of soil samples collected on one-shot sampling was related to: (i) the frequency of finding fox, cat and dog faeces in repeated sampling carried out in the previous months; (ii) the extent of faeces collected from the same sampling; (iii) the detection of *E. multilocularis* in the faeces collected in this sampling, and (iv) the size of the gardens sampled. Because urban gardens are often fenced, but these wooded areas are nevertheless attractive to wild hosts [10, 16], faeces were also collected in the immediate vicinity to the urban gardens.

## Materials and methods

### Study areas

**Figure 1:**
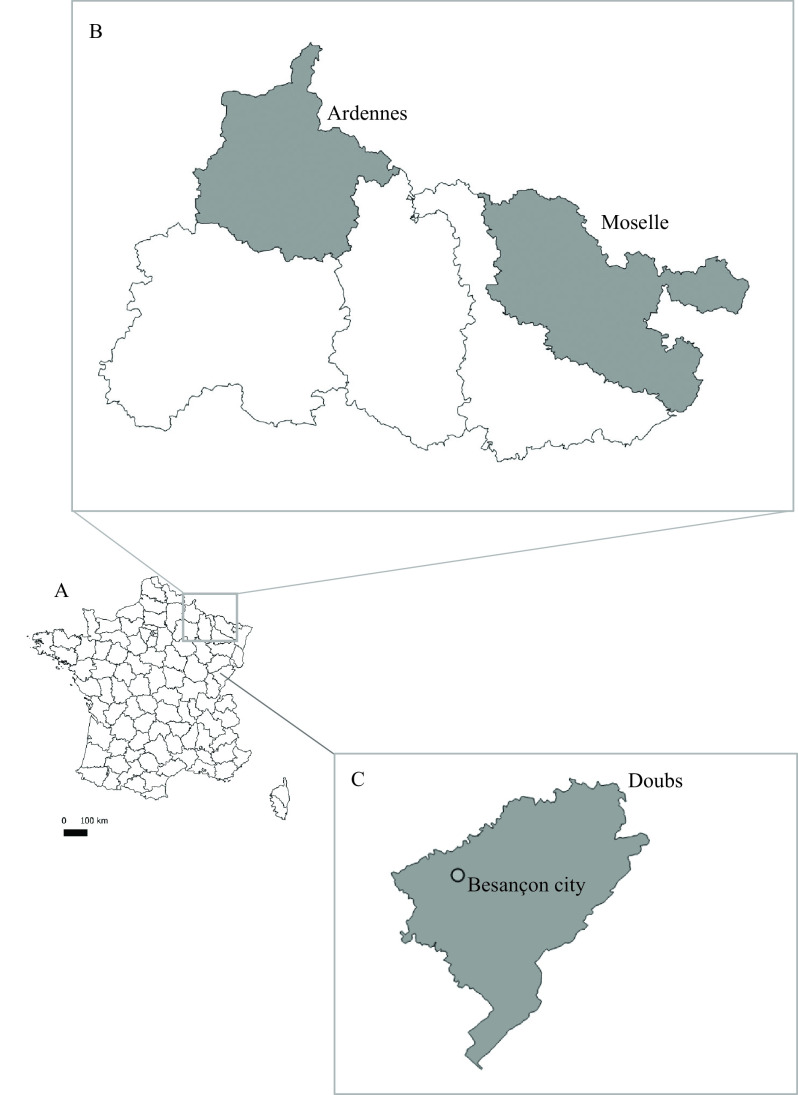
Localisation of the three study areas in France (A), highlighting the rural (B) and urban (C) settings.

The study took place in the three *E. multilocularis* endemic regions of France with the highest AE incidence [42]: Ardennes, Moselle and Doubs (Fig. 1). The French Ardennes (49° 25′ N, 4° 50′ E) is wooded (oak *Quercus* spp., beech *Fagus sylvatica*, hornbeam *Carpinus betulus* and spruce *Picea abies*), with cultivated fields and pastures. The human population density is around 16 inhabitants/km^2^ and most of the villages have fewer than two hundred inhabitants. The Moselle (48° 49′ N, 6° 30′ E) interchanges between wooded and industrialized areas, and human density is around 170 inhabitants/km^2^ and most villages comprise approximately 1000 inhabitants. In the Doubs, the sampling took place in Besançon city (47° 14′ N, 06° 01′ E), half-wooded with forests, parks, vegetable gardens, allotment and worker gardens, and cultivated fields. The human population density is around 366 inhabitants/km^2^.

### Vegetable gardens sampled

In the rural setting (Ardennes and Moselle), 192 vegetable gardens distributed in 38 villages (~4 per villages) were sampled for carnivore faeces during the joint study conducted by Bastien et al. [5]. Among them, 185 were vegetable gardens devoted to household consumption and spanning 207 ± 14 m^2^ in average size (min = 4 m^2^, max = 1276 m^2^). The other seven locations were larger cultivated areas devoted to market gardening and spanning 7550 ± 2275 m^2^ on average (min = 512 m^2^, max = 20,553 m^2^). The total size of these 192 rural vegetable gardens was 91,195 m^2^. In both Ardennes and Moselle, 79% (152/192) of the sampled vegetable gardens were easily accessible to canids because they are unfenced or not effectively fenced [5]. Due to material and financial constraints, only 50 randomly selected vegetable gardens out of the 192 sampled for faeces were also sampled for soil. All were located in the Ardennes: 82% of gardens (41/50) were unfenced or not effectively fenced to prevent canid intrusions. The 50 vegetable gardens sampled for soil were on average 543 m^2^ ± 203 m^2^ in size (min: 15.6 m^2^, max: 8384 m^2^) for a total of 27,176 m^2^. In the urban setting (Besançon city), six cultivated areas surrounded by a minimum 1 m-high fence were sampled: two in the urban centre, two in the city periphery close to wooded areas, and two in an intermediate location. They were on average 6546 m^2^ ± 2891 m^2^ in size (min = 3935 m^2^, max = 10,539 m^2^). Each of them was divided into 20–35 individual vegetable gardens on average 266 m^2^ ± 132 m^2^ in size (min = 178 m^2^, max = 527 m^2^). We sampled between 11 and 12 vegetable gardens per cultivated area, for a total of 71 vegetable gardens and 18,994 m^2^ scanned.

**Figure 2:**
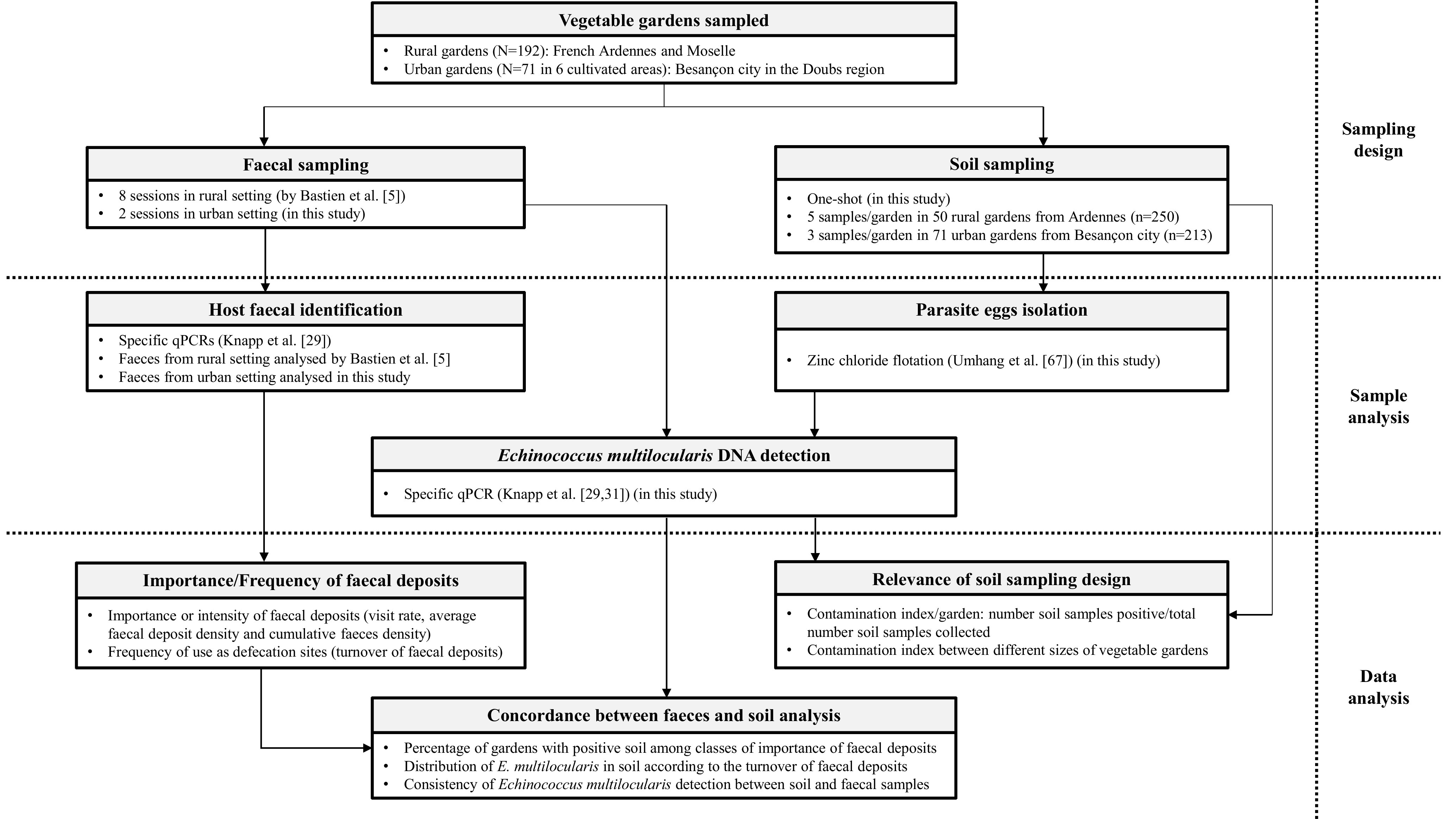
Process chart showing the collection and analysis of faeces and soil samples in rural and urban vegetable gardens located in *E. multilocularis* endemic regions of north-eastern France.

Figure 2 presents a synopsis of the protocol from sampling design to data analysis.

### Faeces and soil sampling

Faeces sampling consisted of visual scans performed by walking the whole surface of the vegetable gardens out of the gardening period to avoid damaging the seedlings. All the carnivore faeces detected during scans were collected and decontaminated over seven days at −80 °C and stored at −20 °C before laboratory analyses. In the Ardennes and Moselle, samplings occurred in February, March, October and December 2014 and in January, March, October and December 2015 for a total of 8 scans per garden (Fig. 2). In the urban setting, faeces sampling occurred twice, in April and September 2019, and was carried out in the 71 selected vegetable gardens and on their access roads and outer edges of the six areas in which they were clustered.

Soil sampling consisted in collecting about 50 g of soil at a maximum of 5 cm from the soil surface. In the rural setting, soil sampling in the 50 vegetable gardens took place in January 2015 and resulted in the collection of five soil samples per vegetable garden, one on each of the four borders and one in the centre, for a total collection of 250 soil samples (Fig. 2). In the urban setting, soil sampling took place in April 2019 in 40 vegetable gardens and in September 2019 in 31 others. For each of the 71 urban vegetable gardens sampled, three soil samples were collected, two at the borders and one in the centre, for a total of 213 soil samples (Fig. 2). All collected soil samples were decontaminated over seven days at −80 °C, and stored at −20 °C before laboratory analyses.

### Molecular analysis

For the faeces, an amount of 0.5 g for each copro-sample was treated for DNA extraction using a QIAamp Fast DNA Stool Mini kit (Qiagen, Hilden, Germany), following the manufacturer’s recommendations. The host faecal test developed as a multiplex real-time PCR test [30] was then used to identify which carnivore species (fox, cat or dog) had released the scat analysed (Fig. 2). The analysis of faeces collected in the urban setting (Besançon city) was conducted in the present study while that of faeces collected in the rural setting (Ardennes and Moselle) was previously conducted by Bastien et al. [5]. For each soil sample collected, 10 g was floated with zinc chloride to concentrate and isolate parasite eggs and the parasite DNA was extracted from the substrate obtained from the filtered supernatant with a QIAamp DNA Mini kit (Qiagen, Hilden, Germany) ([68], Fig. 2). DNA extracts from faeces and soil were then tested in a specific *E. multilocularis* real-time PCR (*E. multilocularis*-qPCR) in order to detect the presence of *E. multilocularis* DNA based on the amplification of a part of the mitochondrial *rrnL* gene ([28, 30], Fig. 2). An internal control tool tested the presence of PCR inhibitors [30]. The two qPCRs were performed in a duplex-qPCR. Reactions with Ct < 45 cycles were considered positive for *E. multilocularis*-qPCR. All samples were tested in duplicate. If at least one of the duplicates was positive, the stool or soil sample was considered positive. If at least one of the three (urban setting) or five (rural setting) soil samples collected in a vegetable garden tested positive, the vegetable garden was considered positive.

### Data analysis

The percentage of fox, dog and cat faeces in the total faeces collection and the occurrence of *E. multilocularis* in faeces were expressed with their 95% confidence intervals (95% CI), and were compared between the 50 soil-sampled gardens and the other 142 rural gardens without soil sampled using Chi-square tests or Fisher’s exact test when sample size was not sufficient.

Three descriptors of the intensity or extent of faecal deposit were computed per vegetable garden and carnivorous species (Fig. 2): (1) **Visit rate** was used as a proxy for the regularity of the vegetable garden use as defecation site. It was calculated as the number of sampling sessions in which faeces were collected relative to the total number of sampling sessions [19, 56]; (2) **Average faecal deposit density** per sampling session was calculated as the number of faeces collected per 100 m^2^ relative to the number of sampling sessions in which faecal samples were found. This descriptor was used as an index of average garden use as defecation site; (3) **Cumulative faecal density** was calculated over the entire sampling as the total number of faeces deposited per 100 m^2^. From these three descriptors, a non-hierarchical cluster analysis following a K-means partition procedure based on Euclidean distance measures was performed to group vegetable gardens according to the extent of faecal deposit [8] defined as “null”, “moderate” or “high”. The values of the three descriptors were first standardized on a 0–1 scale to give the same weight to each garden. Differences in percentage of vegetable gardens with positive soil between these three classes were tested using Chi-square tests of homogeneity.

We assessed the frequency of use of vegetable gardens as defecation sites by determining **faecal density per sampling session,** which was considered an index of the turnover of faecal deposits and was based on the number of faeces deposited per 100 m^2^ every 6 weeks. Faeces collected in December, January and March were taken into account, whereas samples collected in October were not, because they could have been deposited for more than 6 weeks. The influence of the turnover of faecal deposits on the distribution of *E. multilocularis* in the soil of the vegetable gardens (presence/absence) was evaluated using the nonparametric Wilcoxon–Mann–Whitney test. Finally, an index of soil contamination per garden was defined as the number of soil samples positive for *E. multilocularis* eggs DNA on the total number of soil samples collected. The non-parametric Kruskal–Wallis rank sum test was used to compare this contamination index between three classes of garden sizes: small garden (≤150 m^2^), medium garden (151–300 m^2^) and large garden (>300 m^2^). All computations and analyses were performed using R (version 3.5.1 [45]) with significant differences for *p* ≤ 0.05.

## Results

### Faecal contamination of vegetable gardens

At least one scat was found in 148 out of the 192 rural vegetable gardens [5] and in 8 out the 71 urban vegetable gardens sampled, for a total number of 1016 and 8 carnivore faeces collected in the rural and urban settings, respectively (Table 1). Five fox faeces, 6 cat faeces and 9 dog faeces were found at the outer edges of the cultivated areas, outside the fences. Cat, fox and dog faeces accounted for 58.8% (*n* = 597), 31.4% (*n* = 319) and 9.8% (*n* = 100), respectively of the 1016 faeces collected in Ardennes and Moselle [5], whereas of the 8 faeces collected in urban gardens, 3 were attributed to cats and 5 to dogs. The distribution of fox, dog and cat faeces in the rural vegetable gardens did not significantly differ between the 389 faeces collected in the subsampling of the 50 soil-sampled gardens and the 627 faeces collected in the other 142 rural vegetable gardens (Table 1, *p* > 0.05). None of the dog faeces collected tested positive for *E. multilocularis* (Table 1). The distribution of *E. multilocularis* DNA-positive faeces among fox and cat faeces did not vary significantly between the subsampling of the 50 soil-sampled gardens and the other 142 rural vegetable gardens (Table 1, *p* > 0.05). As both sub-samples showed a similar distribution of faeces from different host species and *E. multilocularis* faecal prevalence, data from the 50 soil-sampled rural vegetable gardens were assumed transposable to the entire rural setting studied. At least one fox scat was found in 25 of the 50 vegetable gardens sampled for soil, at least one cat scat was found in 34 of them, and at least one dog scat was found in 12 of them. No faeces collected inside the urban gardens tested positive. However, one out of the five fox faeces collected in the vicinity of the urban vegetable gardens was positive for *E. multilocularis* DNA, as were 18/137 (13.1%) of the fox faeces and 2/224 (0.9%) of the cat faeces collected in the 50 rural vegetable gardens sampled for faeces and soils (Table 1). Faeces that tested positive for *E. multilocularis* were distributed in 12/50 rural soil-sampled gardens (24.0%, 95% CI [13.1–38.2]). Out of the 20 positive faeces, 15 positive fox faeces distributed in 10/50 rural soil-sampled gardens were collected 1–12 months before soil sampling. The two positive cat faeces were found after soil sampling. The importance of faecal deposit in urban vegetable gardens was not assessed due to the low number of faeces collected (*N* = 8). In addition, the relatively small number of dog faeces collected in the rural setting prevented any attempt to analyse their deposition importance. The cluster analysis based on fox and cat faecal descriptors unequally divided the 50 soil-sampled rural vegetable gardens among the three predefined classes (see Table 2 for details of the mean values for each host): (a) 13 had nil faecal deposit; (b) 22 had moderate faecal deposit with an average of 3.42 faeces collected per 100 m^2^ (range 0.11–12.08); and (c) 15 had high faecal deposit with an average of 8.57 faeces collected per 100 m^2^ (range 1.39–57.69). The overall average turnover of faecal deposit was 0.95 per 100 m^2^/6 weeks (range 0.00–12.82), ranging from 0.70 per 100 m^2^/6 weeks (range 0.00–8.55) to 0.25 per 100 m^2^/6 weeks (range 0.00–4.27) for cats and foxes, respectively.

**Table 1:** Number of fox, dog and cat faeces found in rural and urban vegetable gardens in north-eastern France and number of faeces and soil samples testing positive for *E. multilocularis* by real-time PCR.

		Faeces				Soil samples
		Total	Fox	Dog	Cat	
Urban vegetable gardens sampled for faeces and soil (*N* = 71)	No. sampled	8	0	5	3	213
	Positive samples	0	–	0	0	0
	Occurrence %	0	–	0	0	0
	95% CI	–	–	–	–	0-0.02
Rural vegetable gardens sampled for faeces (*N* = 142)	No. sampled	627	182	72	373	–
	Positive samples	35	30	0	5	–
	Occurrence %	5.6	16.5	0	1.3	–
	95% CI	3.9–7.7	11.4–22.7	0–0.1	0.4–3.1	–
Rural vegetable gardens sampled for faeces and soil (*N* = 50)	No. sampled	389	137	28	224	250
	Positive samples	20	18	0	2	26
	Occurrence %	5.1	13.1	0	0.9	10.4
	95% CI	3.2–7.8	8.0–20.0	0–0.1	0.1–3.2	6.9–14.9

95% CI: 95% confidence interval.

**Table 2:** Values for descriptors of fox and cat faeces deposits and proportion of vegetable gardens with at least one soil sample testing positive for *Echinococcus multilocularis* DNA in the three classes of intensity of faecal deposits.

Descriptors (definition)	Species	Intensity of faecal deposit		
		Null	Moderate	High
		*n* = 13	*n* = 22	*n* = 15
**Cumulative faecal density**	Fox	0	1.19 (±0.25)	2.87 (±1.71)
Mean (± SE) number of faeces deposits per 100 m^2^	Cat	0	2.23 (±0.55)	5.69 (±2.16)
	Fox	0	0.66 (±0.17)	1.12 (±0.55)
Mean (± SE) number of faeces deposits per 100 m^2^ relative to the number of sessions with faeces found	Cat	0	1.36 (±0.30)	2.08 (±1.02)
**Visit rate**	Fox	0	0.25 [0.15–0.36]	0.17 [0.07–0.26]
Proportion [95% CI] of sessions with faeces collected relative to the total number of sessions	Cat	0	0.24 [0.17–0.32]	0.38 [0.28–0.45]
**Proportion of vegetable gardens with *E. multilocularis-*positive soil samples**		6/13	6/22	9/15
Percentage [95% CI]		46.2% [20.4–73.9]	27.3% [11.6–50.4]	60% [32.9–82.5]

SE: standard error; 95% CI: 95% confidence interval.

### Soil contamination

**Figure 3:**
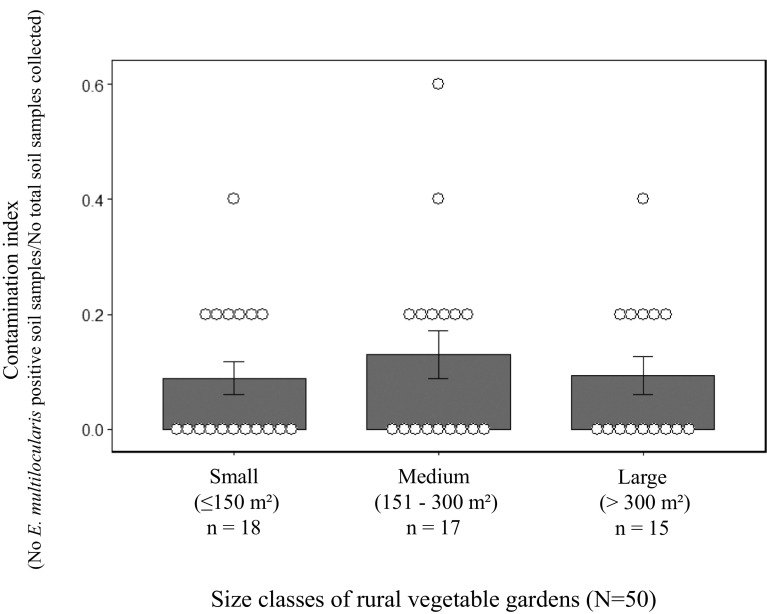
Index of soil contamination by *Echinococcus multilocularis* in relation to the size of rural vegetable gardens sampled in north-eastern France. Bars represent the mean contamination index (± Standard Error); white crenelated dots, the contamination index per vegetable garden.

Of the 250 soil samples from the rural vegetable gardens, 26 (10.4%, 95% CI [6.9–14.9]) tested positive for *E. multilocularis* eggs DNA (Table 1). These *E. multilocularis-*positive soils were distributed in 21/50 vegetable gardens (42.0%, 95% CI [28.2–56.8]). Soil samples tested positive in 4/9 (44.4%) enclosed rural gardens, while no *E. multilocularis*-positive soil was detected among the 71 selected urban vegetable gardens clustered in the 6 cultivated areas – all fenced. In most of the rural vegetable gardens positive for *E. multilocularis* eggs DNA (17/21), only one soil sample tested positive out of the five collected. In three vegetable gardens, 2/5 soil samples tested positive. In one vegetable garden, 3/5 soil samples tested positive. The contamination index did not differ significantly between the small (*n* = 18), medium (*n* = 17) and large (*n* = 15) rural vegetable gardens (Fig. 3, Kruskal–Wallis rank sum test: *χ*^2^ = 0.42, *p* = 0.810).

### Concordance between faeces and soil analysis

**Figure 4:**
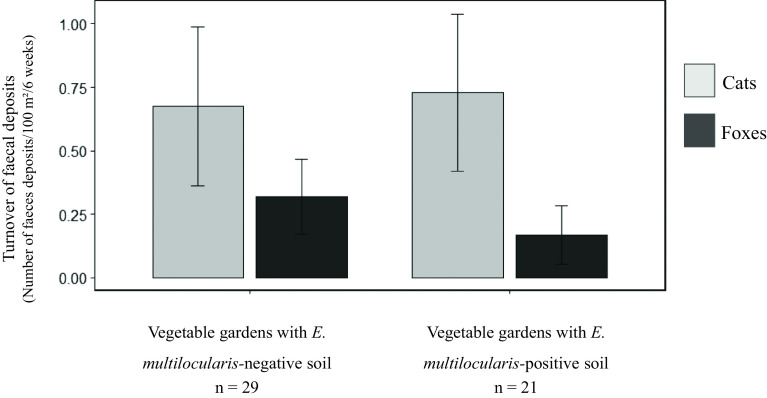
Average turnover of fox and cat faecal deposits in rural vegetable gardens (*N* = 50) sampled in north-eastern France, with soil testing negative and positive for *E. multilocularis* eggs DNA. Line segments represent the standard error.

The turnover of cat and fox faecal deposits in rural gardens did not differ significantly between garden with *E. multilocularis*-negative soil and gardens with *E. multilocularis*-positive soil (Fig. 4, Mann–Whitney test, *W* = 274, *P* = 0.546 and *W* = 395, *p* = 0.056, respectively). Vegetable gardens with at least one contaminated soil sample were homogeneously distributed among those with null, moderate and high faecal deposits (Table 2, *χ*^2^ = 4.05, *p* = 0.132). Specifically, the percentage of gardens with at least one fox scat did not differ between the 21 rural gardens with at least one *E. multilocularis*-positive soil sample and the 29 rural gardens with no positive soils (38.1%, 95% CI [18.1–61.6] *versus* 58.6%, 95% CI [38.9–76.5] respectively, *χ*^2^ = 1.31, *p* = 0.251). Only faeces of cats were found in 6/21 rural gardens where at least one *E. multilocularis*-positive soil sample was collected. Overall, soil and faecal samples both tested positive for *E. multilocularis* in 3/50 rural gardens (6.0%, 95% CI [1.3–16.5]). The percentage of vegetable gardens with at least one *E. multilocularis*-positive soil sample did not significantly differ between the rural gardens where *E. multilocularis*-positive faeces were collected before soil sampling (20.0%, 95% CI [2.5–55.6]) and those where no positive faeces were collected, or where positive faeces were only collected after soil sampling (47.5%, 95% CI [31.5–63.8], Fisher’s exact test, *p* = 0.223). The information provided by the analysis of soil samples collected from rural gardens in January 2015 and that of faeces collected from the same gardens in the previous months is consistent for 21/29 (72.4%) rural gardens in which no *E. multilocularis*-positive samples (either soil or faeces) were found and for 2/21 (9.5%) gardens in which both *E. multilocularis*-positive faeces and soil samples were found (Supplementary Table 1). In the last two cases, the collection of positive faeces (fox faeces in March 2014 and in October 2014) preceded by several months that of a positive soil sample. In 8 rural gardens where at least one positive scat was found at each copro-sampling session (13 positive fox faeces were collected between February and December 2014), no soil tested positive (Supplementary Table 1). In 19 rural gardens where at least one soil sample tested positive, no scat tested positive before soil collection (Supplementary Table 1).

## Discussion

In the urban setting, only a few cat and dog faeces were collected in vegetable gardens and all these faeces tested negative for *E. multilocularis* DNA. It is therefore not surprising that none of the soil samples collected tested positive. However, our urban sampling provides two important pieces of information: the collection of fox faeces on the outer edges but not inside the fenced urban vegetable gardens confirms the effectiveness of fences to prevent fox intrusion [5], and the detection of *E. multilocularis* DNA in one of these faeces attests to the risk of soil contamination by this parasite in the city of Besançon. In the rural setting, *E. multilocularis* DNA was detected in 10.4% of the soil samples collected in vegetable gardens which is consistent with the 11.3% and 11.7% *E. multilocularis*-positive soil samples collected in *E. multilocularis* endemic areas from northeast Poland [59] and north-eastern France [68], respectively. This similar relatively high rate of *E. multilocularis* in soil samples in the latter studies supports the high sensitivity of flotation/filtration techniques combined with molecular biology in the investigation of soil-transmitted parasite. The 35% prevalence of *E. multilocularis* in the local fox population [13] and the use of half of the sampled rural gardens as a defecation site by foxes is sufficient to explain the presence of *E. multilocularis* in the soil of 42% of the 50 rural gardens sampled.

Another interesting finding was the absence of a significant association between the detection of *E. multilocularis-*positive soil samples in the vegetable gardens and the extent and turnover of fox and cat faecal deposit in the 12 previous months. The proportion of vegetable gardens which had at least one fox dropping and one *E. multilocularis* positive soil sample was not higher than the proportion of gardens with no fox dropping but at least one *E. multilocularis-*positive soil sample. In vegetable gardens, frequent watering and turning of the soil can accelerate the degradation of faeces and thus reduce the number of faeces detected [5]. This sampling bias can be reduced by increasing the frequency of sampling as recommended by Sanchez et al. [54].

The fact that only cat faeces were found in six out of the 21 rural vegetable gardens where *E. multilocularis*-positive soil samples were found could be due to insufficient sampling to detect the more rarely deposited fox faeces. However, it is also possible that only cats used these six gardens as defecation sites during the entire study period, in which case the soil would have been contaminated with *E. multilocularis* from cat faeces. The latter finding and the extent of deposit of cat faeces in vegetable gardens [4] even at very low *E. multilocularis* occurrence (less than 1% in this study) support the need for further investigation on the reproductive potential of *E. multilocularis* in cats in the field.

In 19/50 rural vegetable gardens sampled for faeces and soil, *E. multilocularis-*positive soil samples were found while no scat testing positive was previously collected. The persistence of *E. multilocularis* eggs accumulating over time in the soil may explained this result. Such long-term persistence has already been demonstrated for *Echinococcus granulosus* eggs that were found in topsoil samples of plots occupied by experimental infected dogs 41 months after the dogs were removed from the study enclosures, and were found to remain infective after 41 months of aging in soil under environmental conditions of the Patagonia region of Argentina [61, 62].

Insufficient soil sampling may also be responsible for an apparent lack of concordance between faecal deposits and soil contamination. This is probably the reason for the lack of detection of *E. multilocularis* in the five soil samples collected in eight of the rural vegetable gardens where at least one positive fox scat was found at each previous copro-sampling session. Although no significant relationship was found between the detection rate in the five soil samples collected per vegetable garden and the size class of the vegetable gardens (ranging from 15.6 to 8384 m^2^), collecting more soil samples than we did or a given number of soil samples per m^2^ using a systematic method (e.g., systematic aligned or unaligned methods; see [12] for details) may provide a better overview of soil contamination by better taking into account the heterogeneous dispersion of the soil-transmitted helminths in the environmental matrices [12].

In conclusion: (i) the collection and analysis of carnivore faeces allow us to estimate the respective contribution of definitive host species responsible for the contamination of vegetable gardens by *E. multilocularis* and provide an instant indication of potential soil contamination. Regularly screening for the parasite in faeces may also enable us to comprehensively understand the variation in *E. multilocularis* shedding frequency by definitive host species. However, sampling and molecular analysis of faeces do not always reflect the spatial and temporal distribution of soil contamination, even when conducted through repeated sampling; (ii) One-shot soil sampling appears easier to conduct and may provide a better overview of long-term contamination in vegetable gardens where micro-climatic conditions (e.g., regular soil watering) could ensure optimal egg survival and dispersion. However, its reliability depends on the sampling design, which has the advantage of being designed in advance; (iii) Therefore, the analysis of faeces and soil samples should be used in a complementary way. This two-fold analytical approach may provide information on guiding the assessment of the dynamics of environmental contamination by *E. multilocularis* and thus identifying sites at-risk of human contamination. Collection and analysis of more frequent soil samples over a longer time span may provide insights into seasonal and spatial variations in persistence of *E. multilocularis* eggs in soil for relevant estimation of environmental exposure for intermediate hosts and humans. Finally, the presence of *E. multilocularis* positive faeces in vegetable gardens ([41], this study) and the detection of *E. multilocularis* on fruit and vegetables [24, 32] argue for the development of standardized methods allowing for the diagnosis of *E. multilocularis* and other helminths or protozoa on fresh produce. Thus far, no such method is available to estimate the risk of consumer exposure to foodborne parasites in raw fruit and vegetables along the agri-food chain [11, 53].

## Supplementary material

Supplementary material is available at https://www.parasite-journal.org/10.1051/parasite/2021073/olm Supplementary Tables *Supplementary Table 1*. Detailed results of soil and faecal analysis from the 50 soil-sampled rural vegetable gardens.

## Conflict of interest

The authors declare that they have no conflict of interest.
